# Cytotoxic granules and effector molecules from immune cells in tuberculosis: Mechanisms of host defense and therapeutic potential

**DOI:** 10.1080/21505594.2025.2542466

**Published:** 2025-08-18

**Authors:** Yongwei Qin, Jianhao Xu, Qinglan Wang, Jiahai Shi

**Affiliations:** a Department of Pathogen Biology, Medical College, Nantong University, Nantong, China; b Department of Laboratory Medicine, The Yangzhou University Jianhu Clinical College, Jianhu, China; c Department of Respiratory and Critical Care Medicine, Institute of Respiratory Health, West China Hospital, Sichuan University, Chengdu, China; d Department of Thoracic Surgery, Nantong Key Laboratory of Translational Medicine in Cardiothoracic Diseases, and Research Institution of Translational Medicine in Cardiothoracic Diseases in Affiliated Hospital of Nantong University, Nantong, China

**Keywords:** *Mycobacterium tuberculosis*, cytotoxic granules, cytokines, vaccine, immune cells

## Abstract

Tuberculosis (TB), a disease caused by *Mycobacterium tuberculosis* (*Mtb*), remains one of the most formidable infectious diseases globally. The immune system orchestrates a complex response including, but not limited to, T lymphocytes, natural killer (NK) cells, macrophages, and dendritic cells (DCs) to control and eliminate *Mtb*. While these cells are well-recognized for their roles in anti-tumor immunity, their contributions to the defense against *Mtb* are equally critical. This review delves into the specific mechanisms by which these immune cells release cytotoxic enzymes and effector molecules, offering new insights into their pivotal roles in *Mtb* clearance. A deeper understanding of these mechanisms is essential for developing more effective strategies to combat tuberculosis.

## Introduction

*Mycobacterium tuberculosis* (*Mtb*), the causative pathogen of tuberculosis (TB), remains a major global health challenge, causing approximately 10.8 million new infections and 1.25 million deaths in 2023 [1]. *Mtb’*s pathogenic success is largely attributed to its capacity to evade and manipulate the host immune response, establishing a chronic infection that can persist for years. The bacterium primarily targets macrophages, where it resides within phagosomes, evading lysosomal degradation and thus sustaining a latent infection. The host immune system’s response to *Mtb* is a complex and highly coordinated process, characterized by a delicate balance between protective immunity and immunopathology [2].

The host immune response to *Mtb* is an intricate interplay of innate and adaptive immunity. Upon infection, the innate immune system serves as the initial barrier of protection, with macrophages and dendritic cells (DCs) recognizing *Mtb* through pattern recognition receptors (PRRs), initiating a robust pro-inflammatory response. Macrophages, as primary host cells for *Mtb*, deploy various strategies, including the generation of reactive oxygen species (ROS), reactive nitrogen species (RNS), and lysosomal enzymes to fight the infection [3]. Simultaneously, dendritic cells capture *Mtb* antigens and present them to T cells, thereby initiating and modulating the adaptive immune response. DCs are also crucial for inducing antimycobacterial T cell responses through the production of cytokines such as IL-12, IFN-α, and IL-18, which are essential for recruiting and stimulating T lymphocytes and NK cells [4].

**Figure 1.**
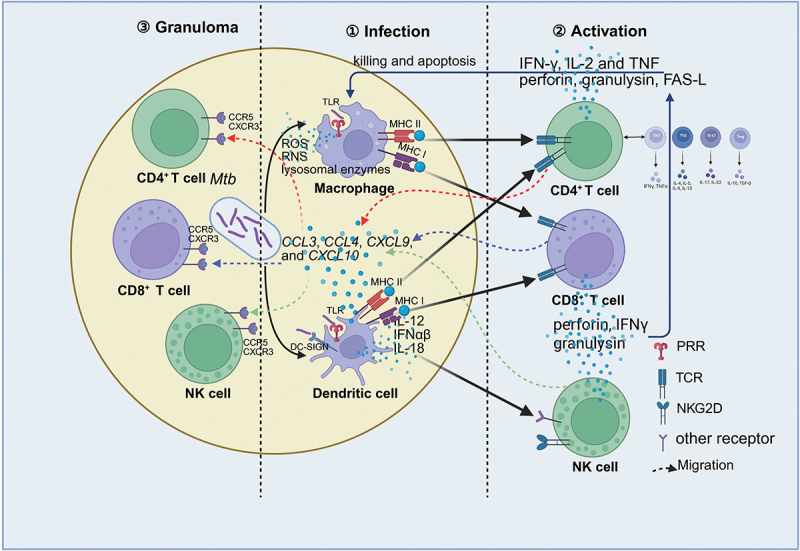
Host immune response to *Mycobacterium tuberculosis*. The host immune response to *Mtb* involves a dynamic interplay between innate and adaptive immunity, ultimately leading to granuloma formation. During the early stages of infection, macrophages and dendritic cells recognize *Mtb* through PRRs, triggering a pro-inflammatory response. Macrophages employ antimicrobial mechanisms such as ROS, RNS, and lysosomal degradation, while DCs process and present *Mtb* antigens to T cells, promoting their activation and cytotoxic function. Activated T cells release perforin and granulysin, which exert direct cytotoxic effects on infected macrophages. Similarly, NK cells release cytotoxic granules to eliminate *Mtb*-infected macrophages. DC-secreted cytokines (IL-12, IFN-α, IL-18) and chemokines (CCL3, CCL4, CXCL9, CXCL10) facilitate the activation and recruitment of NK cells, CD4^+^ T cells, and CD8^+^ T cells, leading to the formation of granulomas that restrict bacterial dissemination. CD4^+^ T cells enhance macrophage bactericidal activity, CD8^+^ T cells mediate cytotoxic responses, and NK cells serve as a crucial bridge between innate and adaptive immunity by killing infected cells and stimulating macrophages. The synergistic interactions among these immune components are essential for controlling *Mtb* infection.

GzmA: Granzyme A; GzmB: Granzyme B; *Mtb: Mycobacterium tuberculosis*; PRR: Pattern Recognition Receptor; RNS: Reactive Nitrogen Species; ROS: Reactive Oxygen Species; TCR: T Cell Receptor; TLR: Toll-Like Receptor; TRAIL: Tumor Necrosis Factor-Related Apoptosis-Inducing Ligand.

The adaptive immune response to *Mtb* relies heavily on the coordinated actions of T cells, particularly CD4^+^ and CD8^+^ subsets, alongside NK cells, macrophages, and dendritic cells. CD4^+^ T cells enhance macrophage microbicidal activity by producing cytokines that activate these phagocytes, whereas CD8^+^ T lymphocytes mediate cytolytic activity against infected targets via the secretion of perforin (PRF1) and granzyme. NK cells function as a crucial bridge connecting innate and adaptive immunity, contributing by directly killing *Mtb*-infected cells and indirectly stimulating macrophages and monocytes. These immune cells do not function in isolation; rather, their complex, interdependent interactions are vital for effectively clearing *Mtb* [5]. This synergy among T cells, NK cells, macrophages, and dendritic cells underpins a robust immune response against the pathogen (Figure 1).

Despite progress in understanding TB immunity, the precise mechanisms governing this cellular coordination remain incompletely understood. This review examines how these immune cells are activated or differentiated in response to *Mtb* infection and identifies the key cytokines and bactericidal products they release to combat the pathogen. By exploring these processes, we aim to clarify their essential roles in TB immunity and propose strategies for developing novel therapeutic interventions and vaccines to address this persistent global health challenge.

## T cell response in *Mtb* infection

### T cell activation or differentiation in response to *Mtb*

CD4^+^ T cells play a crucial role in defending against *Mtb*, but vaccines designed to enhance these responses have had limited success. Studies have shown that during *Mtb* infection, early secretory antigenic target 6 (ESAT-6)-specific T cells become highly differentiated and functionally exhausted due to continuous antigen exposure. In contrast, Ag85B-specific T cells are hindered by reduced antigen expression in persistent infection. These findings suggest that tailored vaccination strategies are necessary to effectively harness T cell responses at different stages of *Mtb* infection [6]. ESAT-6-specific transgenic CD4^+^ T cells, when monitored in vivo, reveal that T cell priming for ESAT-6 begins only after 10 days of infection and is initially restricted to the mediastinal lymph nodes, with subsequent differentiation into proliferative effector cells capable of producing IFN-γ and TNF-α in vitro [7].

Similarly, studies have shown that the adaptive immune response to *Mtb* is characterized by a delayed onset, allowing significant bacterial expansion in the lungs during the pre-immune phase. Adoptive transfer of Ag85B-specific CD4^+^ T lymphocytes has been used to determine that this delay is due to the early delay in CD4^+^ T cell activation, which first takes place in the nearby pulmonary draining mediastinal lymph nodes. Interestingly, initial activation of Ag85B-specific T cells is dependent on the antigen produced by bacteria in the lymph nodes, even though the bacterial count in the lungs is 100 times higher. DCs are responsible for ferrying *Mtb* from the pulmonary environment to regional lymph nodes. This implies that the delayed priming of CD4^+^ T cells during tuberculosis may result from *Mtb* residing in anatomical compartments that are not transferred from the lungs to the lymph nodes [8].

A novel IFN-γ/TNF-independent pathway effectively controls *Mtb* infection. CD4^+^ Th1 lymphocytes targeting the ESAT-6 can suppress bacterial expansion in vivo independently of classical effector mechanisms such as IFN-γ, TNF, perforin, or FAS signaling. In contrast, adoptively transferred Th17 cells reactive to ESAT-6 exhibit moderate control over *Mtb* proliferation, while Th2-polarized CD4^+^ T cells display limited protective capacity [9]. Although TNF production by myeloid cells is important for initial immune responses, it becomes redundant in the later stages of infection. T cells compensate by producing TNF, crucial for maintaining granuloma integrity and modulating the immune response during chronic infection. This underscores the non-redundant role of T cell-derived TNF for long-term protection against *Mtb*, suggesting that therapies targeting TNF should preserve T cell-derived TNF to avoid impairing chronic infection control and potentially exacerbating the risk of reactivation [10]. In macrophages, TNF enhances the immune response to *Mtb* by promoting phagosome maturation [11,12] and apoptosis [13], which, in turn, strengthens T cell responses – a crucial factor for effective TB vaccines. *Mtb* has evolved mechanisms to undermine TNF responses, including biochemical modifications that reduce TNF production [14]. These mutants, through changes in cell surface components and lipid metabolism, exhibit altered virulence and enhanced TNF induction in macrophages, making them promising candidates for vaccine development. Additionally, TNF’s role in promoting CD4^+^ T cell activation and long-lasting memory formation highlights its importance in vaccine efficacy. Vaccination strategies that incorporate TNF and other inflammatory cytokines have shown superior protection and enhanced T cell memory responses in animal models [15]. Future studies will explore whether *Mtb* mutants with enhanced TNF production can induce robust and long-lasting T cell memory to offer protection against TB. In conclusion, regulating TNF production presents a promising approach to enhance the immunogenicity of vaccine candidates.

**Figure 2.**
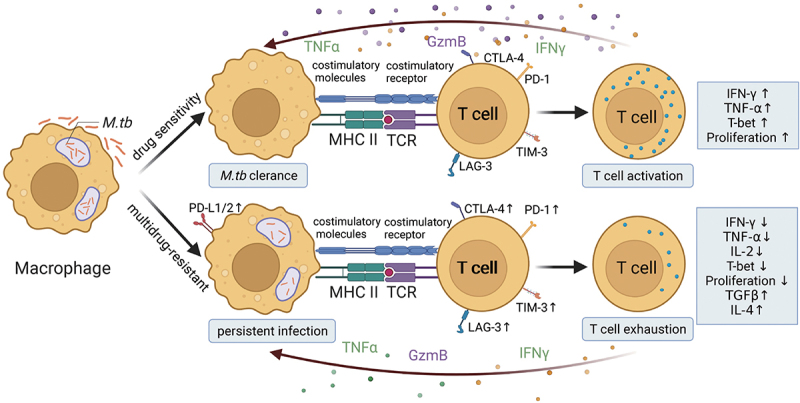
T cell dysfunction and persistent *Mtb* infection in drug-resistant cases. T cells enhance the bactericidal activity of macrophages by secreting cytokines (IFN-γ, TNF-α) and granulysin, facilitating bacterial clearance. In drug-sensitive *Mtb* infections, macrophages successfully eliminate the bacteria, maintaining normal T cell function. However, in multidrug-resistant tuberculosis (MDR-TB) or immunocompromised patients, *Mtb* persists due to immune evasion strategies. Prolonged antigen exposure leads to T cell exhaustion, characterized by diminished cytokine (IFN-γ, TNF-α, IL-2) and granulysin production, impaired proliferation, and increased expression of immune checkpoint molecules (PD-1, CTLA-4, TIM-3, LAG-3). This exhaustion further compromises T cell function and promotes chronic infection. Additionally, persistent *Mtb* infection induces upregulation of PD-L1/2 on infected cells, which suppresses IL-2 secretion and T cell proliferation.

CTLA-4: Cytotoxic T-Lymphocyte-Associated Protein 4; GzmB: Granzyme B; LAG-3: Lymphocyte Activation Gene-3; T-bet: T-box expressed in T cells; TIM-3: T cell Immunoglobulin and Mucin-domain containing-3. ↑up-regulation. ↓down-regulation.

Multidrug-resistant tuberculosis (MDR-TB) strains, such as M and Ra, induce stronger IL-17 responses compared to drug-susceptible strains, particularly in MDR-TB patients. This heightened IL-17 production, associated with CD4^+^ and CD8^+^ T cells, correlates with persistently high antigen loads and cell exhaustion [16] (Figure 2). The immunopathological role of IL-17-producing T cells in MDR-TB may contribute to severe tissue damage and reduced treatment effectiveness of second-line drugs [17]. This phenomenon has been observed in both human and murine models of infection [18] and is characterized by the expression of various immune checkpoints or inhibitory receptors [19], accompanied by a gradual decline in effector functions. Exhausted CD8^+^ T cells exhibit a unique transcriptional profile that inhibits their differentiation into memory or effector cells [20]. This raises the question of whether a specific subset of CD4^+^ T cells exhibits markers of terminal differentiation or exhaustion. The findings display that PD-1-expressing CD4^+^ T cells retain strong proliferative capacity, while KLRG1-expressing cells have a shorter lifespan and produce cytokines such as TNF-α and IFN-γ [21]. Adoptive transfer experiments reveal that proliferating PD-1-positive CD4^+^ T lymphocytes can differentiate into KLRG1-positive cytokine-secreting T cells, and vice versa. This suggests that PD-1^+^ cells are not exhausted but serve a vital role in sustaining the antigen-specific effector T cell response during chronic *Mtb* infection, continuously giving rise to terminal effector cells from a pool of proliferating precursor cells [22]. In addition, persistent infections with *Mtb* may be exacerbated by immunosuppressive mechanisms. Regulatory T cells (Tregs), a subset of CD4^+^ T cells, are crucial for preventing autoimmunity but can also suppress antimicrobial immune responses. Tregs produce immunosuppressive cytokines such as TGF-β and IL-10, thereby dampening CD4^+^ T cell responses and inhibiting the production of pro-inflammatory cytokines. This immunosuppressive milieu can lead to impaired effector immune responses, facilitating *Mtb* dissemination and clinical disease [23]. Post-aerosol infection, Tregs proliferate and accumulate in the pulmonary lymph nodes and lungs. Depletion of Tregs results in a significant reduction in pulmonary bacterial burden, indicating their role in promoting pathological responses to tuberculosis [24].

Despite specific T cell responses, only a small fraction of exposed individuals develops active disease. The differentiation of *Mtb*-specific CD8^+^ T lymphocytes into effector or effector memory cells with low PD-1 expression, even in high bacterial environments is influenced by the host’s inflammatory environment. These cells produce TNF-α and IFN-γ but lack IL-2 production and exhibit limited functional capacity. This limited functionality poses challenges in achieving effective immunity against *Mtb* [25]. The differentiation of CD8^+^ effector cells is a dynamically regulated process that is influenced by the host’s inflammatory environment, resulting in varying phenotypes. By utilizing mixed bone marrow chimeras, a comparison was made between wild-type and cytokine receptor deficient CD8^+^ T cells in the same mouse after *Mtb* aerosol exposure. Four weeks post-infection, it was determined that IL-12, type I IFN, and IL-27 are all essential for the effective expansion of CD8^+^ T cells in the lungs. Utilizing retrovirus-specific CD8^+^ T cells specific for the tuberculosis antigen TB10.4 (EsxH), it was found that IL-12 is the principal cytokine driving the initiation of CD8^+^ T cells in the lymph nodes and their later expansion in the lungs. Yet, type I IFN and IL-27 play distinct roles in promoting the expansion of pulmonary CD8^+^ T cells. Therefore, IL-12 acts as the primary signal for lymph node initiation, but in the lungs, a convergence of multiple inflammatory signals facilitates the continued expansion of pulmonary CD8^+^ T cells. Additionally, these cytokines modulate the differentiation and activity of CD8^+^ T lymphocytes during tuberculosis [26].

### T cell immune responses in tuberculosis: insights into CD4^+^, CD8^+^, NKT, γδ T, MAIT cells and cell function

#### CD4^+^ T cells and CD8^+^ T cells

In patients with active tuberculosis (ATB), an increased frequency of CD4^+^ T cells has been observed, while CD8^+^ T cells display a reduced frequency and exhibit an exhausted phenotype characterized by the expression of CD39, CD279, and TIM-3 [27]. CD4^+^ T cells are crucial in defending against *Mtb* infection by producing cytokines such as IFN-γ and IL-2 that activate macrophages and boost their bactericidal activities. However, patients with tuberculosis often exhibit a weakened Th1 response, with reduced levels of IL-2 and IFN-γ, coupled with a shift towards a Th2 response, marked by elevated IL-4 levels. This shift may inhibit Th1-driven immune responses, potentially impairing the host to combat *Mtb* infection [27]. Single-cell RNA-seq results revealed that in patients with mild and moderate tuberculosis, the expression of IFN-γ was drastically upregulated compared to healthy individuals, while no upregulation was observed in severe TB cases. Similar patterns of IFN-γ expression are seen in NK cells. Additionally, TNF, a key player in granuloma formation and intracellular *Mtb* elimination, shows increased expression in Th1 and NK cells of mild and moderate TB patients but is not upregulated in severe TB patients. The lack of increased IFN-γ and TNF expression in severe TB suggests an impaired immune response in these individuals [28]. In addition to the observed Th1 responses in patients with active tuberculosis, the functional characteristics of T cells in the granuloma environment also provide significant insights. Granuloma T cells showed enrichment in Th1 cell-associated genes (e.g., CXCR3, CCR6, RORC), highlighting their Th1/Th17-like phenotype. The study found a significant role for CD161, CD40L, and IL-26 in protective immune responses, as their expression inversely correlated with bacterial loads. The study also reveals the critical role of the CD153 axis in T cell-mediated immunity, where CD30 expression on CD4^+^ T cells is essential for effective host resistance against *Mtb* infection. These findings contribute to understanding T cell responses in TB and could inform vaccine development strategies [29]. In line with these findings, studies indicate that *Mtb* manipulates host immune responses by promoting Th2 cell differentiation, facilitated by the RD-1 and ESAT-6 of the bacterium, leading to the production of IL-1β. This IL-1β not only drives Th2 responses that counteract protective Th1 immunity but also shows a dual role in either promoting protective innate responses or aiding in disease progression by inducing Th2 cells. These findings suggest that disrupting Th2 and regulatory T cell (Treg) induction could enhance vaccine efficacy against tuberculosis [30].

Additionally, CD4^+^ T cells have been found to possess cytotoxic T lymphocyte (CTL) features, indicated by increased expression of perforin and elevated transcriptional levels of granulysin, granzyme A (GzmA), granzyme B (GzmB), and perforin, along with the expression of T-bet (Tbx21) and NKG2D (Klrk1). These cytotoxic characteristics are more pronounced in drug-sensitive tuberculosis (DS-TB) compared to drug-resistant tuberculosis (DR-TB). Proteomic analyses have further identified the presence of heat shock protein 70 (HSP70) in DS-TB and annexin A5 in DR-TB, which may support the cytotoxic CTL features in these patients [27].

Although antigen-specific effector CD4^+^ T cells can restrict *Mtb* bacterial growth, they are insufficient to prevent initial infection establishment. This delay in controlling bacterial growth despite the presence of effector T cells suggests that persistent tuberculosis might be attributed more to the failure in the activation of these antigen-specific T cells rather than their development. Moreover, research demonstrates that enhancing T cell stimulation – by increasing antigen availability – can improve long-term infection outcomes without causing detrimental effects. Notably, even a moderate increase in antigen-specific CD4^+^ T lymphocyte activation significantly reduces bacterial burden [31]. Future tuberculosis therapies should focus on strategies to enhance effector T cell activation, addressing barriers such as direct T cell inhibition, reduced antigen presentation, and bacterial strategies that limit immune responses.

#### NKT cells

In tuberculous pleurisy, pleural fluid – resident CD3^+^ TCRvβ11^+^ NKT cells exhibit an activated effector memory phenotype and contribute to local antimycobacterial immunity through both cytokine production and cytotoxic activity. Numerous glycolipids stimulate NKT cells and CD1-restricted T cells to secrete IFN-γ, thereby triggering a swift innate immune response [32]. Invariant NKT (iNKT) cells exert antimycobacterial effects by secreting IFN-γ, which activates infected macrophages to produce nitric oxide through inducible nitric oxide synthase (iNOS). In addition to IFN-γ, iNKT cells express cytotoxic mediators such as perforin and Fas/FasL, enabling them to kill Mycobacterium tuberculosis within infected macrophages. Their activation depends on CD1d-restricted recognition and is further enhanced by IL-12 and IL-18 signaling [33,34]. These combined mechanisms allow iNKT cells to suppress bacterial replication and contribute to early innate immune defense. These findings highlight the dual role of NKT cells in orchestrating inflammatory and cytolytic responses against *Mtb* at the site of infection.

#### γδ T cells

γδ T cells represent an unconventional subset of T lymphocytes that have garnered increasing attention in immunotherapy research. In healthy adults, they comprise only 1–5% of peripheral blood lymphocytes and express a T cell receptor (TCR) composed of γ and δ chains, which exhibit limited diversity and enable recognition of non-peptide antigens independent of classical major histocompatibility complex (MHC) restriction or antigen-presenting cell (APC) involvement [35]. While the roles of CD4^+^ and CD8^+^ αβ T cells in mediating protective immunity against *Mtb* infection are well established [36,37], accumulating evidence over recent years has increasingly implicated γδ T cells as important contributors to host defense against *Mtb* [38,39].

During persistent *Mtb* infection, NK-like CD8^+^ γδ T cells exhibit enhanced cytotoxicity by releasing cytolytic granule components including GzmB, perforin, and IFN-γ. These cells also express CD16 (FcγRIIIa), enabling antibody-dependent cellular cytotoxicity (ADCC). Their granule exocytosis machinery includes NKG7, and they produce additional cytotoxic mediators such as GzmA, GzmH, and granulysin (GNLY). This polyfunctional profile – marked by co-expression of GzmB, PRF1, and IFN-γ—enables these γδ T cells to control intracellular *Mtb* effectively, despite their diminished TCR responsiveness [40]. Upon stimulation with specific *Mtb*-HAg components – particularly HspX, GroEL1, and GroES—γδ T cells, predominantly of the Vγ2 Vδ2 subset, produce elevated levels of TNF-α and IFN-γ. Although GzmB expression was not directly quantified, the cytotoxic effect of stimulated γδ T cells on *Mtb*–infected THP-1 cells suggests the involvement of granule-mediated mechanisms. This functional profile confirms that γδ T cells exert their protective effect through TNF-α, IFN-γ, and likely perforin/granzyme pathways [41].

Human γ9δ2 T cells confer protective immunity against *Mtb* through multiple cytotoxic and immunomodulatory mechanisms. A key effector molecule is GzmA, which is secreted independently of perforin and acts by stimulating infected macrophages to produce proinflammatory cytokines, particularly TNF-α and IL-1β—critical mediators for suppressing intracellular *Mtb* replication. Notably, GzmA alone is sufficient to induce these responses, and its expression strongly correlates with the antimicrobial capacity of γδ T cells. Silencing GzmA significantly impairs the ability of γ9δ2 T cells to control *Mtb* infection, underscoring its central role. This represents a non-classical cytotoxic mechanism that does not rely on perforin, apoptosis, autophagy, or nitric oxide production [42]. In addition to GzmA, activated Vγ9 Vδ2 T cells also secrete perforin and granulysin that mediate direct killing of intracellular *Mtb* within infected macrophages. Furthermore, these cells upregulate Fas and Fas ligand (FasL) upon antigenic stimulation, promoting target cell apoptosis and contributing to activation-induced cell death (AICD), thereby maintaining immune homeostasis during infection [43].

#### Mucosal-Associated Invariant T (MAIT) cells

Mucosal-Associated Invariant T (MAIT) cells are another subset of T cells involved in the immune response to *Mtb*. These cells are highly prevalent in humans and are enriched at mucosal sites, including the airway - where *Mtb* first interacts with the host. MAIT cells may serve as diagnostic indicators for *Mtb* infection due to their innate ability to detect bacterially derived riboflavin metabolites presented by the MR1 molecule. Moreover, the presence and functional profile of MAIT cells in peripheral blood and tissues could reflect recent antigen exposure or immune clearance, offering translational relevance in distinguishing latent, active, or cleared TB infection [44,45].

MAIT cells facilitate the initiation of *Mtb*-specific CD8^+^ and CD4^+^ Th1 responses, which are critical for controlling tuberculosis. Notably, CD8^+^ MAIT cells in individuals exposed to *Mtb* exhibit higher reactivity, suggesting their pivotal role in the clearance of initial *Mtb* infection [44]. Additional studies have revealed new functions for CD4^+^ and CD8^+^ MAIT cells in early *Mtb* exposure. CD8^+^ MAIT subsets in IGRA^+^ contacts demonstrate higher reactivity, while IGRA^−^ contacts show lower CD69 expression and CD25 induction upon MR1 ligand stimulation. Increased expression of GzmB in MAIT cells from IGRA-negative contacts, in the absence of an IFN-γ response, suggests that GzmB^+^ MAIT cells may serve an important role in targeting and responding to *Mtb*-infected cells during the early stages of infection [46,47].

MAIT cells show therapeutic potential for tuberculosis due to their ability to recognize *Mtb* antigens and produce pro-inflammatory cytokines (e.g., IFN-γ, TNF-α, IL-17A), which enhance antimycobacterial immunity. In murine models, artificial boosting with 5-A-RU and co-stimuli increased MAIT cell abundance by 40–80-fold in lung and liver tissues, suggesting a role in controlling bacterial infections [48]. In chronic *Mtb* infection, 5-OP-RU administration reduced bacterial burden via IL-17 induction, indicating that MAIT cells may adopt an antimicrobial phenotype [49]. In rhesus macaques, MAIT cells expanded and migrated to infection sites during active and latent TB, eliciting a rapid Th1 response, and redifferentiated MAIT-like cells (reMAIT) demonstrated effective pathogen control in *Mycobacterium abscessus* infection, reducing bacterial load by 40–50% through granulysin release [50,51]. These findings suggest MAIT cells could enhance host protection and inform novel TB immunotherapies [52]. However, significant limitations challenge their translational potential in primates. In rhesus macaques infected with *Mtb*, intratracheal 5-OP-RU administration failed to yield clinical or microbiological benefits, as MAIT cells upregulated PD-1, exhibited exhaustion, and lost the ability to produce cytokines (IFN-γ, TNF-α, IL-17A). Vaccination with 5-OP-RU plus CpG in uninfected macaques similarly impaired function without expansion, and PD-1 blockade only partially restored cytokine secretion without rescuing proliferation. These results indicate that MAIT cells in primates are prone to dysfunction following TCR stimulation, posing a major barrier to their use in TB vaccine or therapeutic strategies [53].

Beyond conventional MAIT cells, a specialized subset known as MAIT NKT cells has emerged as a key player in the early immune response to *Mtb*, further highlighting the diversity and complexity of T cell-mediated antimycobacterial defenses. Their hybrid gene expression signatures, resembling both activated NK cells and conventional CD8^+^ T cells, support their function as a bridge between innate and adaptive immunity. Elevated levels of MAIT NKT cells were observed in subjects with latent tuberculosis infection (LTBI), particularly those who progressed from LTBI to active pulmonary TB, suggesting their involvement in early disease control. However, when MAIT NKT cells fail to contain the infection, sequential recruitment of CD16^+^ CD56^dim^ and CD16^−^ CD56^bright^ NK cells may occur, indicating a compensatory response [54]. This dynamic underscores the temporal coordination between NKT and NK cell-mediated immunity in tuberculosis pathogenesis.

### The dual-edged sword of cytotoxic factors in pulmonary tuberculosis

Recent research has highlighted the importance of cytokine production by T cells in controlling *Mtb* infection. Nonconventional memory CD8^+^ T cells and NK cells produce IFN-γ in an *Mtb* antigen-independent manner, regulated by IL-18 and the bacterial survival sensing mechanism, playing a crucial role in host defense against *Mtb* infection. This antigen-independent production of IFN-γ is critical for reducing mortality and morbidity in *Mtb*-infected hosts. In patients with tuberculosis, particularly those with severe disease, elevated expression of these cytotoxic factors has been observed. These findings provide mechanistic insights into antigen-independent IFN-γ secretion and its potential application in developing new tuberculosis interventions [55].

While the antigen-independent production of IFN-γ plays an important role in host defense, the cytotoxic mechanisms mediated by perforin, granzymes, and granulysin further increase the immune response against *Mtb*, highlighting both their protective and potentially pathogenic effects in tuberculosis. Pulmonary tuberculosis patients exhibit increased expression of GzmA in CD4^+^ T cells, CD8^+^ T cells, and CD56^+^ T cells, but not in NK cells. Moreover, significant upregulation of GzmB has been observed in CD8^+^ T cells compared to controls. Although increased extracellular levels of GzmA – and to a lesser extent GzmB – have been observed, they do not correlate with intracellular levels within lymphocyte subsets. These findings highlight the significant upregulation of GzmA and GzmB in both intracellular and extracellular environments in TB patients [56]. Granulysin, expressed by activated CD8^+^ T cells, NK cells, and γδ T cells, exhibits broad antimicrobial activity against *Mtb*, including drug-resistant strains. Its high expression in tuberculosis patients, particularly in CD4^+^ T cells with a CTL phenotype, is associated with increased production of perforin and granzymes, further enhancing the cytolytic activity against *Mtb* [57]. Studies have shown elevated percentages of perforin-positive CD8^+^ T cells in active pulmonary TB patients, suggesting increased cytolytic activity against *Mtb* [58]. However, chronic TB is linked to decreased production of perforin and granulysin in CD8^+^ T cells at the infection site, potentially contributing to disease progression [59]. The importance of perforin-2 for bacterial killing is further emphasized by the finding that heterozygous MPEG1 mutations in humans are associated with persistent nontuberculous mycobacterial infections, leading to reduced antibacterial activity of neutrophils, macrophages, and B cells [60]. These observations underscore the critical role of Perforin-2 in protecting against bacterial infections and its potential involvement in both acute and chronic infectious diseases.

**Figure 3.**
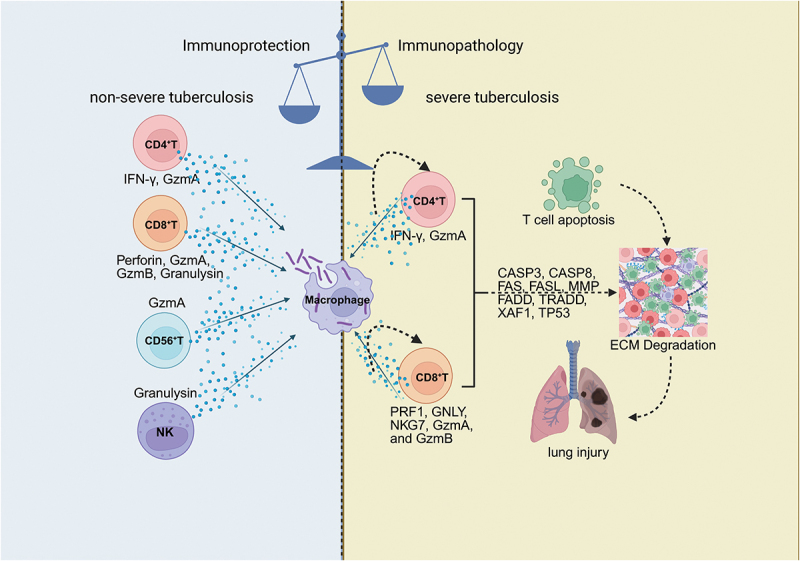
In tuberculosis (TB), immune cells such as CD4^+^ T cells, CD8^+^ T cells, NK cells, and macrophages coordinate a defensive response by releasing cytotoxic molecules such as IFN-γ, granzymes, and granulysin to kill *Mtb*. While these immune responses are crucial for pathogen clearance, excessive cytotoxicity in severe TB can lead to tissue damage and immune dysfunction. Overexpression of cytotoxic proteins results in increased apoptosis and disruption of tissue integrity. In contrast, non-severe TB shows a more controlled immune response, effectively clearing the bacteria. This balance between protection and pathology is critical for understanding TB immunity and improving treatments.

CASP3: Caspase 3; CASP8: Caspase 8; FAS: Fas Cell Surface Death Receptor; FASL: Fas Ligand; MMP: Matrix Metalloproteinase; FADD: Fas-Associated Protein with Death Domain; GzmA: Granzyme A; GzmB: Granzyme B; TRADD: TNFRSF1A-Associated via Death Domain; XAF1: XIAP Associated Factor 1; TP53: Tumor Protein p53

However, excessive cytotoxicity may exacerbate tissue damage in patients with severe tuberculosis (Figure 3). Overexpression of cytotoxic genes in effector CD8^+^ T cells and NK cells, such as NKG7, GNLY, PRF1, GzmA, and GzmB, may contribute substantially to immunopathology. Upregulation of cytotoxic effector proteins, including granzymes, may lead to organ damage by degrading the extracellular matrix and triggering inflammatory responses [28,61]. In patients with severe tuberculosis, heightened expression of perforin and granzymes in T and NK cells has also been associated with increased apoptosis. This immunopathology is further supported by the upregulation of apoptotic markers, including CASP3, CASP8, and FAS. Specifically, activation of the extrinsic apoptotic pathway (involving FAS, FASL, FADD, TRADD, and CASP8), along with XAF1-mediated TP53-dependent apoptosis, appears to be active in both CD4^+^ and CD8^+^ T cells. These findings suggest that multiple apoptotic mechanisms contribute to the immune dysregulation observed in severe tuberculosis [28]. Moreover, stimulation with *Mtb* antigens enhances the expression of CD137 and CD137L on immune cells, thereby modulating both innate and adaptive immune responses. Notably, blocking CD137 increases IFN-γ and TNF-α production by monocytes and NK cells, while simultaneously reducing CD8^+^ T cell degranulation and cytokine production, and enhancing T cell apoptosis [62].

In summary, T cells, particularly CD4^+^, CD8^+^, NKT, γδ T and MAIT cells, are integral to the immune response against *Mtb*. They exert their effects through cytokine production, cytotoxic granule release, and interactions with other immune cells. However, the delicate balance between protective immunity and immunopathology underscores the complexity of T cell-mediated responses in TB. Understanding these dynamics is essential for developing targeted therapies that enhance protective immunity while minimizing tissue damage.

### T cell exhaustion and cytotoxic granule dynamics: implications for immunotherapy

In tuberculosis lung tissues, clonally expanded CD8^+^ and CD4^+^ T cells exhibit reduced expression of exhaustion markers (e.g., PDCD1, TIGIT, LAG3, SNX9, PELI1) and exhaustion-related transcription factors (e.g., TOX, GATA3, BATF, RUNX2, PRDM1), indicating impaired T cell exhaustion compared to tumor and non-tumor lung tissues [63–66]. Notably, expanded CD8^+^ effector/memory (Teff/mem) and exhausted (Tex) T cells in TB lungs show elevated expression of cytotoxic granule GzmK, IFN-γ and TNF, suggesting heightened inflammatory and cytotoxic activity [64,66], while other cytotoxic granules (e.g., GzmM) and activation-related transcripts (e.g., S100A4, S100A6) are downregulated in both CD8^+^ and CD4^+^ Teff/mem cells [66]. Precursor exhausted T cells (Texp) in TB lungs, primarily CD8^+^ Tem, share similar characteristics, with high GzmK, TNF, and IFNG expression and reduced PDCD1, indicating an activated, non-exhausted state that may drive immunopathology rather than effective *Mtb* control [63,67]. Additionally, TB lungs are enriched with CMV-reactive CD4^+^ and CD8^+^ T cells, particularly Tem, which may contribute to inflammation through bystander activation or cross-reactivity with *Mtb* antigens. This process may be driven by CMV-induced ZNF683 expression [63,68–71]. *Mtb*-reactive CD4^+^ T cells, predominantly Tem and Tcm, show significant clonal expansion, while *Mtb*-reactive CD8^+^ T cells are less abundant and minimally expanded, suggesting differential roles in TB immunity [72].

Targeting exhaustion markers like PD-1 to restore anti-*Mtb* immunity, inspired by cancer immunotherapy, has shown detrimental effects in TB. In mouse and rhesus macaque models, anti-PD-1 therapy exacerbated TB, increasing bacterial loads and pro-inflammatory cytokine production (e.g., IFN-γ, TNF) by *Mtb*-specific CD8^+^ T cells. At the same time, CD4^+^ T cell trafficking to granulomas was reduced [73,74]. The reduced exhaustion in clonally expanded T cells, particularly CD8^+^ Tex and Texp cells with high GzmK expression, may contribute to this exacerbated pathology by driving non-resolvable inflammation [63,67,75]. This mirrors findings in atherosclerosis, where GzmK^+^ CD8^+^ T cells with reduced exhaustion also promote inflammation, suggesting a shared mechanism that anti-PD-1 therapy may amplify [63,76,77]. The presence of CMV-reactive T cells in TB lungs further complicates therapeutic targeting, as their expansion may enhance inflammation without effectively controlling *Mtb* [68]. These findings indicate that while reduced T cell exhaustion and elevated cytotoxic granule release (e.g., GzmK) may enhance immune activation, they risk exacerbating TB pathology, highlighting the need for cautious therapeutic strategies and further studies to target chronic inflammation without compromising immune regulation.

## NK cell response in *Mtb* infection

NK cells, potent innate lymphocytes, are essential in the early defense against *Mtb* infection, particularly in immunocompromised individuals, where their cytolytic abilities are crucial for lysing *Mtb*-infected monocytes and enhancing mycobacterial resistance. NK cells exert their influence on *Mtb* growth through direct cytotoxicity and immune modulation by producing perforin, granulysin, granzymes, IFN-γ, and IL-22, which facilitate phagolysosomal fusion, macrophage activation, and γδ T cell proliferation. These cytotoxic mechanisms, well-documented against tumor cells and intracellular bacteria, are also employed by NK cells to target and kill intracellular bacterial pathogens such as *Mycobacterium kansasii* and *Mtb*. The killing mechanism is contact-dependent and involves the stimulation of cell surface receptors and intracellular signaling cascades, resulting in heightened production of a series of cytotoxic granules [78].

### NK cell recognition and activation of mycobacterial components

During bacterial infections, various pathogen-derived ligands activate Toll-like receptors (TLRs) and pattern recognition receptors (PRRs), inducing specific inflammatory responses. This indirect activation of NK cells, leading to IFN-γ production, can enhance macrophage phagocytosis of infected cells or extracellular bacteria. Recent studies have shown that cell wall-derived proteins from *Mycobacterium bovis BCG*, *Pseudomonas aeruginosa* and *Nocardia farcinica*, can directly engage NKp44, potentially contributing to NK cell activation. Interestingly, blockade of NKp44 did not inhibit NK cell activation, suggesting that its upregulation might be a consequence of activation rather than its initial trigger, possibly amplifying the response. Additionally, cell wall components such as α-glucan (AG) and peptidoglycan (PG) from mycobacteria are known to interact with both NKp44 and TLR2 receptors on NK cells. Engagement of TLR2 with mycobacterial components has been shown to activate resting NK cells and stimulate IFN-γ production. Although the interaction between NKp44 and its ligands appears to play a secondary role, it may contribute to maintaining prolonged NK cell activation [79]. Marcenaro et al. demonstrated that isolated human NK cells could directly recognize *M. bovis BCG*, triggering activation, cytokine secretion, and target cell lysis, a response inhibited by TLR2 blocking antibodies, indicating a functional TLR2 receptor on NK cells [80]. However, the specific bacterial ligands and NK cell receptors involved remain to be fully identified, contrasting with other studies that highlight the importance of signals from accessory cells in NK cell stimulation by BCG [81].

### Cytotoxic factors and immune modulation by NK cells

Upon non-specific IL-12 stimulation, NK cells from TB patients showed significantly lower expression of CD69, CD107a, and intracellular IFN-γ compared to healthy controls. However, when stimulated with *Mtb*-specific antigens, NK cells from TB patients exhibited a marked increase in CD69, CD107a, and IFN-γ expression compared to those from healthy donors. These findings suggest that, while NK cell activation, degranulation capacity, and cytokine production are generally diminished in TB patients, *Mtb*-specific antigen stimulation can significantly enhance NK cell function [82].

NK cells from both PPD-negative and PPD-positive subjects are capable of killing intracellular *Mtb*, demonstrating that this effect is not solely dependent on prior exposure to tuberculosis. The mechanism of killing does not involve IFN-γ production or the transfer of cytotoxic substances through supernatant, but rather requires direct cell-to-cell contact between NK cells and *Mtb*-infected monocytes. NK cells may induce apoptosis in *Mtb*-infected monocytes through alternative pathways, distinct from Fas/FasL interactions [83]. We have also explored whether NK cell-mediated killing of *Mtb*-infected cells depends on cytotoxic granules, such as granzyme, or death receptor-ligand interactions like TNF or TRAIL. One study highlighted that NK cells can first kill target cells via granule-mediated cytotoxicity and subsequently switch to death receptor-mediated killing. This transition reflects a temporal shift in NK cell cytotoxicity – from early GzmB/perforin-mediated killing to later death receptor-mediated apoptosis during repeated encounters with target cells. The interplay between GzmB and death receptor pathways remains complex but is crucial for serial NK cell killing. In particular, GzmB-mediated killing was shown to require perforin, while death receptor-mediated killing could occur independently of perforin, underscoring the differential regulation of these cytotoxic pathways during NK cell serial killing [84].

As previously discussed, NK cells in tuberculosis patients exhibit altered activation and cytotoxic responses, particularly upon stimulation with *Mtb*-specific antigens. Further research has revealed that patients with active tuberculosis (aTB) show a reduction in the total number and proportion of NK cells compared to those with latent tuberculosis infection (LTBI). Specifically, aTB patients have fewer CD56^neg^ NK cells expressing early maturation markers like CD161, NKp30, and NKp46. However, these patients exhibit higher densities of NKp46 and NKp30 expression on CD56^neg^ and CD56^dim^ NK cell subsets, correlating with increased GzmA and GzmB expression, which play a key role in the elimination of infected macrophages and intracellular bacteria [85].

In addition to these findings, the HIV-induced metabolite δ-Aminolevulinic acid (ALA) inhibits the production of IFN-γ in NK cells, as well as the expression of NF-κB, AP-1, and antimicrobial peptides, while simultaneously disrupting glycolysis in NK cells within the context of *Mtb* infection. The ALA-induced reduction in glycolysis further promotes autophagy in NK cells, leading to a further decrease in IFN-γ secretion. Elevated ALA levels during HIV infection promote the dissemination of *Mtb* and the advancement of the disease. Additionally, NK cells can destroy *Mtb*-infected monocytes and alveolar macrophages via NKp46 and NKG2D receptors, thus boosting CD8^+^ T cells’ capacity to generate IFN-γ and clear *Mtb*-infected cells [86].

Turning to newly diagnosed pulmonary TB patients, NK cell subsets are often found to be reduced, along with a decrease in the expression of activating receptors NKp30 and NKp46, which are important for the induction of perforin and granulysin [87]. Antituberculosis treatment regimens have been shown to partially restore NK cell cytotoxicity by reducing the *Mtb* burden, and glutathione (GSH) can also partly suppress intracellular *Mtb* growth via bacteriostatic processes. These findings highlight a novel role for NK cells, perforin, and granulysin in combating mycobacterial infections and suggest an alternative immune defense strategy [88]. In cavitary TB patients, NK cells showed higher expression of GzmA and GzmB, rather than perforin, upon *Mtb* antigen stimulation, leading to apoptotic cell death. The study suggests that the elevated baseline transcription of perforin may facilitate granzyme entry into target cells, amplifying the cytotoxic response [89].

Moreover, research has recognized a subset of NK cells expressing the CD27 marker, indicative of memory or mature NK cells, which plays a pivotal role in controlling *Mtb* infection. In macaques and humans, CD27^+^ NK cells expand in response to mycobacterial stimulation, producing higher levels of PRF1 and GzmB, which are critical for their cytotoxic potential against TB infection [90].

Further research has identified a specific subset of NK cells that express a set of classic cytotoxic genes (Prf1, Ncr1, GzmA, GzmB, Fcer1g) and killer cell lectin-like receptors (Klra8, Klrb1c, Klre1), which are important for initiating signaling and promoting Th2 responses. This subset of cytotoxic NK cells was found to be present in the lungs of mice, with their proportion decreasing from 16.7% in uninfected lungs to 10.3% at 100 days post-infection with *Mtb*. This indicates a decline in the population of cytotoxic NK cells as *Mtb* infection becomes chronic and the disease advances [91].

In summary, NK cells, upon stimulation by *Mtb*-specific antigens, upregulate activating receptors and secrete significant amounts of perforin and cytotoxic granules. These granules trigger apoptosis in target cells through two main mechanisms: interaction between death receptors and their ligands, and perforin-mediated entry of granzymes into the target cell. Once inside the target cell, granzymes activate caspases and cleave specific substrates to trigger apoptosis. Importantly, NK cells avoid self-damage during this process by translocating CD107a from cytotoxic granules to their external membrane, which inhibits perforin binding to the NK cell surface [92]. Furthermore, by forming pores on the membranes of target cells, perforin facilitates the entry of cytotoxic molecules into *Mtb*-infected cells, thereby exerting direct bactericidal and clearance effects. However, the precise roles of granule-associated enzymes such as granulysin and perforin in host cell and intracellular *Mtb* elimination remain poorly understood, underscoring the necessity for continued investigation into their cytolytic pathways.

## Macrophages: central players in *Mtb* control

### Differentiation and activation of macrophage response to *Mtb* infection

Upon infection with *Mtb*, macrophages undergo a complex activation and differentiation process, which is crucial for effective immune responses to *Mtb*. The activation state of macrophages, influenced primarily by IFN-γ, determines whether *Mtb* proliferates or remains latent. IFN-γ activates signaling cascades such as the JAK-STAT and NF-κB pathways, enhancing macrophage antimicrobial capacity through increased nitric oxide (NO) and reactive oxygen intermediate (ROI) production. Targeting these pathways presents potential strategies for enhancing host defense against tuberculosis [93].

Monocytes recruited to the lungs and peripheral lymph nodes (PLN) undergo distinct differentiation pathways. In the lungs, monocytes can become classically activated Mφ, critical for controlling bacterial growth. Alveolar macrophages act as the first line of defense, recognizing and phagocytosing the bacteria. This response includes the secretion of microbicidal products and orchestration of T cell responses. However, *Mtb* has evolved mechanisms to subvert these defenses. Early in infection, ESAT-6, a key *Mtb* effector, drives macrophages towards a pro-inflammatory M1 phenotype, promoting granuloma formation and enhancing the recruitment of immune cells. Over time, *Mtb* manipulates macrophages to switch from the M1 to an immunomodulatory M2 phenotype, which facilitates the establishment of a “silent” granuloma with slow-growing mycobacteria. This transition not only aids in immune evasion but also supports chronic infection by creating an environment conducive to long-term persistence [94]. Conversely, within the PLN, monocytes predominantly differentiate into mature dendritic cells, expressing high levels of CD86 and CD83 without acquiring a macrophage-like phenotype [95]. These findings highlight the dynamic nature of monocyte differentiation driven by tissue-specific cues and inflammatory signals, revealing their pivotal role as progenitor cells for diverse immune cell types essential for orchestrating effective immune responses during tuberculosis. Additionally, *Mtb* regulates foam cell formation to enhance its persistence and evade host immune responses. During TB granuloma formation, *Mtb*-infected alveolar and interstitial macrophages transform into foam cells, characterized by lipid accumulation. The metabolic reprogramming of macrophages involves excessive glycolysis and defective mitochondrial respiration, leading to lipid accumulation and foam cell formation [96]. ATF2 influences metabolic reprogramming and enhances macrophage antibacterial activity against *Mtb* by modulating glycolysis, promoting M1 polarization, and improving antigen presentation [97].

Moreover, *Mtb* disrupts the normal maturation process of monocytes into fully functional macrophages and dendritic cells, leading to impaired cytokine production, diminished T cell activation, and reduced antigen-presenting capability. These less differentiated mononuclear phagocytes exhibit functional deficiencies, such as decreased expression of key surface markers (e.g., HLA-II, CD86) and reduced responsiveness to T cell cytokines like IFN-γ. The resultant impaired immune responses suggest that *Mtb* exploits these suboptimal host cells to evade immune surveillance and enhance its persistence. This mechanism underscores the potential for targeting monocyte and macrophage maturation pathways as a strategy to improve tuberculosis treatment and control [98].

### Cytotoxic factor release and bacterial killing by macrophages

Macrophages release several key antimicrobial factors and cytokines to combat *Mtb*. The primary cytokine involved is interferon-gamma (IFN-γ), which activates several crucial pathways. IFN-γ induces the production of IL-15 and IL-32, leading to the activation of CYP27B1 and the transformation of 25-hydroxyvitamin D into its biologically active metabolite, 1,25-dihydroxyvitamin D₃ (1,25(OH)₂D₃). This active vitamin D stimulates the expression of antimicrobial peptides such as cathelicidin and DEFB4 [99,100]. Additionally, 1,25(OH)₂D₃ triggers autophagy, facilitating the fusion of phagosomes with lysosomes, thereby enhancing the degradation of *Mtb*. This pathway helps overcome the *Mtb*-induced block in phagosome maturation and promotes the killing of bacteria. Furthermore, autophagy is essential for delivering antimicrobial peptides and ubiquitinated peptides to *Mtb*-containing vesicles, thus contributing to microbial killing. Vitamin D sufficiency is crucial for this IFN-γ-induced antimicrobial response; vitamin D deficiency impairs the production of these antimicrobial peptides and reduces the effectiveness of the immune response against *Mtb*. This mechanism highlights the importance of vitamin D in enhancing both innate and adaptive immune responses to tuberculosis and highlights the potential for vitamin D supplementation in improving TB treatment and prevention [101].

Activated macrophages produce ROS via NADPH oxidase (NOX2) and RNS through inducible nitric oxide synthase (NOS2), which act to damage microbial components and inhibit bacterial survival. However, *Mtb* has evolved mechanisms to counteract these defenses; for example, the *Mtb* protein Eis modulates autophagy and inflammatory responses to enhance survival, while proteins like Ndk and nuoG interfere with NOX2 and ROS production. Additionally, *Mtb* employs protective enzymes to neutralize ROS, adapting to harsh conditions within granulomas by sensing environmental changes through specific proteins. Thus, *Mtb*’s ability to evade oxidative and nitrosative stress is a key factor in its persistence and virulence within the host [102].

In addition to intrinsic antimicrobial responses, the extracellular lung environment also modulates macrophage activity. For instance, Alveolar Lining Fluid (ALF) hydrolases modify *Mtb* cell wall components, releasing fragments that enhance macrophage control of *Mtb* through IL-10 and specific transcription factors, challenging the previously held notions that IL-10 promotes *Mtb* growth. ALF-exposed *Mtb* fragments improve macrophage phagolysosome fusion and acidification, despite not affecting *Mtb* recognition. The study reveals that these fragments influence macrophage function, suggesting that the lung environment significantly impacts the infection outcome by modulating macrophage responses to *Mtb* [103].

Interestingly, studies using alternative model organisms such as zebrafish further corroborate the importance of macrophage priming. Pre-treatment with heat-killed Listeria monocytogenes enhances *Mtb* control through upregulation of key immune genes such as macrophage-expressed gene 1 (mpeg1), TNF-α, and NOS2b, indicating that prior immune priming enhances macrophage-mediated control of *Mtb* [104].

### Cytotoxic factor release and macrophage cell death

While macrophages play a defensive role against *Mtb*, their own cell fate is tightly manipulated by the pathogen. Notably, non-classical monocytes (CD16^+^) exhibit a heightened susceptibility to apoptosis following *Mtb* infection. Studies have shown that apoptosis scores were significantly higher in severe TB-infected individuals. This increased apoptosis is linked to the upregulation of apoptosis-related genes such as FADD, TNFSF10, FAS, TNFRSF10A, and TNFSF12, which are associated with the FAS, TNF, and XAF1 pathways, suggesting that these pathways may contribute to elevated monocyte apoptosis in severe TB cases [28]. *Mtb* has developed strategies to subvert host cell death mechanisms to favour its survival and persistence, which play a vital role in infection outcomes. Early in infection, *Mtb* inhibits apoptosis, while later stages induce necrotic cell death [105]. Apoptosis is associated with a protective host response, whereas necrosis favours pathogen survival and dissemination. Various cell death modalities, including pyroptosis, necroptosis, and autophagy, are involved in *Mtb* infection [106]. The interplay between host and bacterial proteins is critical for pathogenesis, with several mycobacterial factors modulating multiple cell death pathways [107]. This complex interplay between *Mtb* and host cell death mechanisms continues to be a focus of tuberculosis research.

Apoptosis, characterized by caspase activation and minimal inflammation, often leads to the containment and clearance of *Mtb* within apoptotic bodies through efferocytosis. Necrosis, driven by RIPK1/3 and MLKL, and pyroptosis, triggered by caspase-1 and gasdermin D, involve intense inflammatory responses that can lead to rapid pathogen clearance. *Mtb*, however, often avoids these processes by inhibiting inflammasome activation and altering host cell death mechanisms, allowing it to persist and potentially spread. The interaction between *Mtb* and macrophage death pathways is complex and strain-dependent, with *Mtb* employing various strategies to subvert immune responses and ensure its survival within the host [102]. Recent studies reveal a novel mechanism in which apoptotic cells infected with *Mtb* release extracellular vesicles carrying mycobacterial antigens to uninfected bystander antigen-presenting cells (APCs). These vesicles facilitate the activation of CD8^+^ T cells through MHC-I and CD1b pathways, bypassing the need for direct cytoplasmic antigen processing, which is otherwise limited due to sequestration in phagosomes. Although infected macrophages no longer retain the ability to display antigens and stimulate CD8^+^ T cells, uninfected dendritic cells can effectively stimulate CD8^+^ T cells when added to infected macrophage cultures. This process underscores the role of apoptosis in tuberculosis as a critical step for cross-priming CD8^+^ T cells [108]. Exosomes secreted by macrophages infected with intracellular pathogens like *M. tuberculosis*, *Salmonella*, and *Toxoplasma* can stimulate proinflammatory responses in macrophages. The research identifies that these exosomes, containing PAMPs such as LPS and 19-kDa lipoprotein, trigger TNF-α production through TLRs. Exosomes from *M. bovis BCG*-infected cells also elicited responses but with partial activity in TLR2- and TLR4-deficient macrophages, indicating the presence of multiple functional PAMPs [109]. The results indicate that apoptotic vesicles and exosomes derived from infected cells serve as important sources of antigens for MHC-I-mediated cross-presentation, providing insights into designing new vaccines that target both CD4 and CD8^+^ T cells.

Beyond antigen presentation, *Mtb*-infected macrophages secrete soluble factors that induce T cell apoptosis in a dose-dependent and contact-independent manner. This T cell death is mediated by factors larger than 50 kDa, which are heat-labile and distinct from known apoptotic pathways involving TNF-α and Fas/Fas Ligand. These factors disrupt the immune response by depleting T cells near the infection site, potentially inhibiting the activation of macrophages and allowing *Mtb* to evade destruction and persist within the host. This mechanism highlights the complex strategies employed by *Mtb* to manipulate host immunity and facilitate its survival [110].

Cathepsin B (CTSB), which is essential for the activation of the NLRP3 inflammasome and subsequent production of the cytokine IL-1β. IL-1β is critical for host resistance against *Mtb* but can also contribute to immunopathology if produced in excess. The *Mtb* virulence factor ESAT-6 is instrumental in promoting CTSB activity, facilitating the escape of the bacteria from the phagosome into the cytosol, and inducing necrotic macrophage death. This process triggers inflammasome assembly and IL-1β maturation, which are crucial for an effective immune response. However, excessive IL-1β production, linked to high CTSB activity, can lead to detrimental inflammation, highlighting the dual role of these factors in both protective and pathological responses during *Mtb* infection [111].

## Dendritic cells: bridging innate and adaptive immunity

### Maturation and antigen presentation by dendritic cells in response to *Mtb*

The activation, maturation, and differentiation of dendritic cells following *Mtb* infection are indispensable for coordinating an effective anti-tuberculosis immune response. Dectin-1 involvement in *Mtb*-infected immature monocyte-DCs facilitates the induction of mature DCs, which produce TNF-α, IL-1β, IL-6, and IL-23. These mature DCs, in turn, direct CD4^+^ cells to secrete IFN-γ and IL-17, promoting Th1/Th17 responses. The cytokine release induced by *Mtb* in DCs is dependent on the participation of the Dectin-1 receptor, conversely, activation via the Dendritic Cell-Specific Intercellular adhesion molecule-3-Grabbing Non-integrin (DC-SIGN) or mannose receptor (MR) suppresses this process [112]. *Mtb* binds significantly more to DC-SIGN-expressing cells compared to cells lacking DC-SIGN. This binding is not restricted to laboratory strains, as similar results were observed with clinical isolates. DC-SIGN was shown to be the major receptor for *Mtb* on human monocyte-derived DCs, even in the presence of complement, while other receptors like CR3 and MR played a minor role. The binding of *Mtb* was inhibited by mannose-rich ligands such as mannan and *Mtb*-derived lipoarabinomannan (LAM), suggesting that LAM is a key ligand for DC-SIGN. DC-SIGN is rapidly excluded from *Mtb*-containing phagosomes, indicating a potential recycling mechanism. In vivo, DC-SIGN^+^ cells in lymph nodes from tuberculosis patients were found to harbor mycobacterial material, reinforcing the idea that DC-SIGN mediates *Mtb* interaction during natural infection [113]. Building on the role of DC-SIGN in *Mtb* recognition, it is also crucial to understand how other cellular interactions influence DC maturation and the overall immune response.

Similarly, the interaction between dendritic cells and *Mtb*-infected polymorphonuclear neutrophils (PMNs) via the DC-SIGN and Mac-1 receptors is essential for effective DC maturation. Studies have shown that blocking these receptors disrupts DC development, underscoring the importance of this cell –cell communication. Interestingly, DC maturation does not appear to be solely dependent on integrin expression levels on infected PMNs, but rather by the co-localization of multiple stimulatory ligands. Additionally, the study suggests that DC-SIGN might facilitate proximity for other ligand-receptor interactions rather than being the primary signaling pathway for DC activation. The findings highlight the underestimated role of PMNs in tuberculosis immunity, not only in bacterial killing but also in initiating specific immune responses through DC maturation after PMN apoptosis. Future research should focus on further elucidating the mechanisms of innate and adaptive immune system crosstalk and its impact on tuberculosis immunity, particularly in enhancing DC-mediated T cell responses [114]. In addition to cellular interactions, the functional activation of DCs is further enhanced by Reactive oxygen species (ROS) production, which is critical for antigen processing. ROS production, driven by TLR2 and dectin-1, enhances DC functionality by promoting antigen processing and presentation, which is essential for effective T cell activation. The study reveals that *Mtb*-induced ROS facilitates DC maturation, including the up-regulation of CD86 and HLA-DR, and is necessary for both non-specific and antigen-specific T cell proliferation [115].

Another significant antigen involved in DC activation is the *Mtb* PPE60 protein, which has been shown to promote Th1 and Th17 responses through a TLR2-dependent mechanism. The PPE60 protein of *Mtb* promotes Th1 and Th17 immune responses via a TLR2-dependent mechanism by inducing DC maturation and NLRP3 inflammasome activation, leading to enhanced production of IL-1β and IL-18. This process drives CD4^+^ T cells to differentiate into Th1 and Th17 cells, characterized by increased production of IFN-γ and IL-17A. The findings suggest that PPE60’s ability to stimulate DCs and activate the NLRP3 inflammasome could be pivotal in developing effective TB vaccines [116]. Understanding how *Mtb* antigens can enhance the immune response provides insight into vaccine development strategies. Supplementation of the BCG vaccine with *Mtb* antigens such as ESAT-6 and HspX significantly enhances DC activation, resulting in increased cytokine production, CD4^+^ T cell priming, and NK cell stimulation. This is evidenced by elevated IFN-γ levels and CD69 expression [117].

In addition to specific antigens, the cell wall components of *Mtb* also influence the functional properties of DCs during infection. The impact of *Mtb* strains with varying cell wall components, particularly α-glucans on dendritic cell function and subsequent immune responses. *Mtb*’s ability to persist within host cells and evade immune detection is influenced by its surface polysaccharides and glycolipids. Notably, α-glucans in the *Mtb* capsule play a crucial role in modulating DC maturation and function. Strains lacking α-glucans, like *Mtb*, induce less reactive oxygen species (ROS) in DCs, impairing their maturation and T cell activation capabilities. This study highlights that α-glucans facilitate ROS production and DC maturation via Syk signaling, enhancing the immune response. Therefore, the presence of α-glucans appears essential for effective DC-mediated immune responses against tuberculosis [118].

Another consideration in *Mtb*’s immune evasion is how irradiated bacteria affect DC differentiation and immune responses. Research reveals that irradiated *Mtb* disrupts DC differentiation from monocytes *in vitro*, potentially aiding in immune evasion. Irradiated *Mtb* induces the loss of a significant portion of monocytes and alters the differentiation of the remaining cells, leading to an enrichment of a DC subpopulation with reduced CD1b expression and diminished ability to stimulate specific T cell proliferation. This altered differentiation resembles features seen in DCs from patients with active tuberculosis, suggesting that *Mtb* may evade the immune response by impairing DC maturation, which compromises the initiation of effective *Mtb*-specific T cell responses [118].

Despite the immune evasion mechanisms employed by *Mtb*, DCs remain crucial in initiating T cell responses, as demonstrated by their phagocytic activity. DCs are instrumental in initiating T cell responses by capturing and presenting antigens. When cultured from peripheral blood and exposed to *Mtb*, human DCs effectively phagocytose the bacteria, leading to increased expression of MHC class I (MHC-I) molecules and costimulatory molecules (CD54, CD40, B7.1), and secrete inflammatory cytokines such as TNFα, IL-1α, IL-1β and IL-12 [119,120]. In contrast, *Mtb*-infected human monocytes do not show enhanced costimulatory molecule expression but do secrete cytokines, indicating that *Mtb* directly activates and matures DCs, enhancing their ability to present antigens and stimulate T cells [121]. Moreover, it has been shown that apoptotic macrophages containing *Mtb* antigens can significantly enhance DC maturation and T cell activation. A comparable study also demonstrated that DCs developed a mature phenotype, marked by upregulated expression of MHC-I, MHC-II and costimulatory markers such as CD40 and CD86, thereby boosting CD8^+^ T cell responsiveness and promoting IFN-γ secretion. Apoptotic macrophages containing mycobacterial antigens, induced by mycobacterial lipoprotein LpqH, facilitated this maturation, unlike UV-induced apoptotic macrophages, which lacked such antigens and led to a different DC phenotype. DCs showed increased production of both proinflammatory (TNF-α, IL-12) and anti-inflammatory (IL-10) cytokines, though IL-10 predominated [122]. The study highlights that DCs efficiently cross-present antigens from apoptotic bodies, requiring proteasome activity and endosomal acidification, and suggests that the incorporation of mycobacterial antigens within apoptotic bodies plays a pivotal role in facilitating this mechanism.

Finally, beyond antigen presentation, DCs also play a pivotal role in shaping the overall immune response by promoting Th1 polarization. Unlike macrophages, which are involved in granuloma formation and exhibit significant proinflammatory and immunosuppressive responses, DCs specialize in antigen presentation and T cell activation, thus playing a critical role in shaping the adaptive immune response and potentially informing vaccine strategies against tuberculosis [123]. *Mtb*-activated DCs can induce protective immunity in mice. The conditionally immortalized DC line tsDC and primary bone marrow-derived DCs (BMDCs) effectively phagocytose *Mtb*, control bacterial growth, and activate protective immune responses. This protective effect is attributed to the DCs’ ability to up-regulate cytokine, prime T cell responses, and potentially involve cross-presentation of mycobacterial antigens. Two injections of *Mtb*-infected DCs were found to generate immunity comparable to or better than BCG vaccination, highlighting the potential of targeting DCs for enhanced vaccine strategies against tuberculosis [124].

### Cytokine and toxic factor release by dendritic cells

DCs and macrophages, despite both being antigen-presenting cells (APCs), exhibit distinct responses to *Mtb* infection. While both cell types internalize *Mtb* and can inhibit its replication through nitric oxide synthase (NOS2)-dependent mechanisms, only macrophages can effectively kill intracellular bacteria. DCs, despite similar levels of reactive nitrogen intermediates (RNI) production, do not reduce bacterial numbers over time. DCs show superior ability to stimulate T cell proliferation and IFN-γ production, which may be crucial for priming effective T cell responses. However, *Mtb* may persist within DCs, potentially aiding in its dissemination and evasion of immune responses. This study suggests that *Mtb* might exploit DCs for transport to lymph nodes, highlighting the need for targeted vaccine strategies that address DC-mediated immunity [125].

Infected DCs efficiently polarize naïve T cells into Th1 effectors, primarily through IL-12 secretion, despite concurrent IL-10 production. The presence of IL-10 does not inhibit Th1 differentiation, likely due to the rapid induction of IL-12. In contrast, macrophages infected with *M. tuberculosis* primarily produce IL-10 and initially fail to secrete IL-12; however, they can produce IL-12 in the presence of IFN-γ, which also down-regulates IL-10. This differential cytokine response suggests that DCs are crucial for initiating Th1 responses early in the infection by transporting antigens to lymph nodes and secreting IL-12, while macrophages play a role in maintaining the Th1 response and microbicidal activity within the granuloma. The findings highlight the unique roles of these APCs in the immune response to *Mtb* and suggest that targeting DCs might be advantageous for vaccine development and improving Th1 responses in tuberculosis [126]. In addition to their role in Th1 differentiation, DCs also engage in Th17 pathway modulation, IL-23 and IL-17 play critical roles in DC responses to mycobacterial infections. Th17 cells play a crucial role in inflammation by secreting cytokines that recruit inflammatory immune cells like neutrophils and monocytes, to the infection site. Cytokines such as IL-17, IL-17F, IL-21, and IL-22, further promote the synthesis of antimicrobial peptides such as defensins [28]. When DCs are infected with *Mtb* or *M. bovis* BCG and present the antigen to CD4^+^ T lymphocytes, the presence of IL-23 during initial priming is necessary for the efficient induction of Th17 cells [127]. Moreover, following low-dose aerosol *Mtb* infection, γδ T cells become major producers of IL-17, a process supported by IL-23 signaling. The IL-17 produced by these cells promotes the formation and maturation of granulomas, thereby enhancing the sequestration and elimination of *Mtb* [128,129]. Specifically, TB-Multiple Antigen Presenting System (MAPS) vaccination promotes the expansion of lung-resident γδ T cells capable of secreting IL-17A and GzmB upon antigen stimulation. IL-17A facilitates granuloma formation and recruitment of other immune cells, while GzmB enhances direct cytotoxicity against infected cells. This dual functionality enables γδT cells to support both inflammatory signaling and pathogen elimination within the pulmonary microenvironment [130]. In addition to IL-17-mediated responses, γδ T cells produce interferon-γ (IFN-γ) in response to IL-12, whereas NKT cells preferentially secrete IFN-γ upon IL-23 stimulation. This differential cytokine responsiveness reflects their unique roles in the early immune response and underscores the importance of both IL-12 and IL-23 signaling pathways in orchestrating IFN-γ–mediated antimycobacterial defense. Furthermore, cross-regulation between IL-17 and IFN-γ may influence dendritic cell function and shape the overall inflammatory outcome during mycobacterial infection [131,132].

Following *Mtb* infection, DCs generate key chemokines that orchestrate the immune response [133]. Specifically, CCL3 and CCL4 are implicated in modulating leukocyte recruitment and potentially down-regulating CCR5 expression on DCs, while CXCL9 and CXCL10 are crucial for directing Th1/Tc1-activated lymphocytes and NK cells to the infection site. The production of CXCL10 is notably regulated by type I IFN and NF-κB pathways, with IFN-αβ being a central mediator of its induction. CXCL10 secretion initiates around 8 hours post-infection, coinciding with the timing of DC migration to lymphoid organs, thereby facilitating a targeted immune response [134].

Despite effective TB treatments, patient non-compliance and drug resistance pose significant challenges. The study introduces N2T4 as a novel approach to enhance host immunity and improve the efficacy of TB drugs. N2T4 treatment led to increased activation and functionality of DCs, enhanced autophagy, and improved migration to lymph nodes. This resulted in elevated production of cytokines, augmented T cell memory responses, and a notable reduction in bacterial survival. Importantly, combining N2T4 with TB drugs like isoniazid (INH) and rifampin (RIF) allowed for lower drug dosages while maintaining efficacy, thereby reducing side effects and potentially mitigating drug resistance. This approach presents a promising strategy for more effective TB treatment by boosting host immune responses alongside traditional therapies [135].

Expanding on this therapeutic approach, identifying the molecular regulators of DC function offers further insight into host-directed TB therapies, as drug-resistant strains of *Mtb* and the protracted development of new therapies highlight the need for alternative strategies, research has shifted towards enhancing host immune responses. This study focused on identifying host genes crucial for mediating immune responses to *Mtb* via dendritic cells. Using RNAi libraries, several genes were identified and involved in the calcium calmodulin and cysteine protease pathways that significantly impact *Mtb* survival and immune modulation. Notably, genes like *Usp25*, *Snrk*, and *Senp8* were found to negatively regulate proinflammatory responses and bacterial survival. These findings reveal that these genes modulate autophagy, oxidative bursts, and cytokine production, thereby influencing the immune response to *Mtb*. This understanding of gene function could enhance strategies to combat TB by targeting immune regulation mechanisms [136].

## Cooperative interactions among T cells, NK cells, macrophages, and DCs

The immune response to *Mtb* infection is not a solitary event involving independent actions of individual immune cells. Understanding and elucidating the intricate network of interactions and cooperation among various immune cells within the tuberculosis-infected microenvironment is crucial for developing effective immunotherapeutic strategies against tuberculosis.

The interplay between NK cells and other immune components is critical in orchestrating a robust defense against *Mtb*. IL-21, secreted by *Mtb*-activated CD4^+^ T cells, enhances NK cell function, driving the secretion of IFN-γ and antimicrobial peptides that target *Mtb*-infected monocytes. These activated NK cells also boost CD8^+^ T cell responses, modulate monocyte cytokine production – increasing IL-1β, MIP-1β and IL-18, while reducing IL-10—and secrete IL-22, which restricts *Mtb*’s intracellular growth. Moreover, IL-21 synergizes with IL-2 to amplify NK cell activity, including the elimination of *Mtb*-expanded CD4^+^ regulatory T cells, underscoring the pivotal role of NK cells in both innate and adaptive immunity [137]. Additionally, NK cells exert regulatory effects through interactions with other innate immune cells. For instance, they interact with CD11c^+^ cells, leading to elevated IL-6 production in the lungs during *Mtb* infection, particularly in type 2 diabetes mellitus (T2DM) contexts [138]. This finding highlights the role of NK cells in modulating immune responses in the context of both infectious and metabolic diseases, offering insights into the complex interplay between these cells and their environment during *Mtb* infection [138]. Notably, NK cells secrete IFN-γ in response to *Mtb* independently of B cells, T cells, and monocytes, highlighting a direct interaction between CD56^bright^ NK cells and *Mtb*, possibly involving other accessory cells at the infection site [139]. In severe TB, however, this coordinated immune response is disrupted. Patients exhibit a dramatic shift in immune cell composition, characterized by a significant increase in monocytes (up to 90% of PBMCs) and megakaryocytes. Conversely, there’s a substantial depletion of lymphocytes, including NK cells, CD4^+^ T cells, CD8^+^ T cells, MAIT cells, and γδ T cells, indicative of lymphopenia. This imbalance in immune cell populations is associated with the severity of TB infection and is accompanied by lymphopenia [28]. In severe TB, lymphocyte reduction is likely triggered by the activation of multiple apoptosis pathways such as FAS, TNF, perforin/granzyme, and XAF1. This condition is further defined by a state of immune exhaustion that impacts Th1, CD8^+^ T cells, and NK cells, and is coupled with an increase in cytotoxic activity in the same cell populations [28].

NK cells interact intricately with neutrophils to mitigate inflammation during *Mtb* infection, and their depletion can impair NK cell function, reflecting their interdependence. They contribute to DC maturation and T cell activation through direct interactions or IFN-γ production, enhancing anti-TB immunity by promoting γδ T cell and *Mtb*-specific CD8^+^ T cell proliferation. Additionally, NK cells modulate adaptive immunity by cytotoxic effects on immature DCs and Tregs, balancing the effector and regulatory arms of the immune response [140]. Collectively, these interactions underscore the multifaceted role of NK cells in controlling TB, including their capacity to enhance CD8^+^ T cell responses, eliminate CD25^+^ regulatory T cells, and directly lyse extracellular *Mtb* [87].

In addition to NK cells, emerging evidence indicates that neutrophils support the priming of naïve, antigen-specific CD4^+^ T cells by promoting the transit of *Mtb* from pulmonary sites to the draining lymph nodes. This is achieved through the transfer of bacteria to dendritic cells, which then present antigens more effectively, enhancing T cell activation and proliferation [141]. This neutrophil-DC cooperation underscores the significant role of neutrophils beyond their traditional innate functions, positioning them as key contributors to adaptive immunity.

Despite these complex and coordinated immune defenses, a major challenge in TB vaccine development lies in the delayed activation of T cells. This is not due to the failure to induce strong T cell responses but rather due to delays in antigen presentation and T cell activation. By targeting dendritic cells to enhance their function, such as through DC transfer or activating specific DC pathways, researchers can accelerate vaccine-induced T cell responses, leading to more effective early control of *Mtb* infection. This approach overcomes a critical bottleneck in vaccine efficacy by rapidly initiating T cell responses and macrophage activation, thereby improving protection against TB. The findings suggest that improving vaccine strategies should focus on overcoming delays in T cell activation rather than solely enhancing T cell induction [87]. Using the pCD11c-DTR/GFP Tg mouse model, the study demonstrates that DCs are crucial for priming CD4^+^ T cell responses to *Mtb* infection, particularly for generating ESAT-6-specific T cells. DCs are essential for initiating adaptive immunity and controlling bacterial replication, as their depletion delays T cell response and impairs infection control. However, once primed, CD4^+^ T cell recall responses do not depend on DCs, suggesting other antigen-presenting cells can activate memory T cells. These findings underscore the critical role of DCs in early immune responses to *Mtb* and their impact on infection outcomes [142]. Thus, optimizing DC function represents a critical leverage point for improving TB vaccine efficacy.

Prolonged or excessive antigen exposure can drive T and NK cells into a state of immune exhaustion, characterized by upregulation of inhibitory receptors such as Tim-3, PD1, BTLA, CD200, and KLRG1 [28]. This exhaustion results in diminished cytokine production, particularly of IFN-γ and TNF-α, together with increased levels of transcription factors associated with T cell exhaustion, such as Blimp-1. In severe TB patients, exhausted NK cells, Th1, and CD8^+^ T cells secrete reduced levels of IFN-γ, a critical cytokine for macrophage activation and bacterial clearance. IFN-γ is crucial for facilitating phagosome maturation and enhancing the generation of reactive oxygen and nitrogen species, which are essential for effective bacterial killing [143]. Additionally, recent findings indicate that IFN-γ promotes autophagy in macrophages by engaging TLR, IRGM1, and PI3K signaling cascades, thereby facilitating the trafficking of ubiquitinated cargo to lysosomes and enhancing their antimicrobial function [144].

Macrophages and dendritic cells internalize *Mtb*, process its antigens, and present them via MHC class II molecules to CD4^+^ T cells. In parallel, they secrete IL-12, which drives Th1 differentiation, leading to IFN-γ production that reinforces macrophage antimicrobial functions such as reactive oxygen/nitrogen species (ROS/RNS) generation and autophagy [145,146]. Activated CD4^+^ T cells further contribute by secreting IFN-γ, IL-2, and TNF, which in turn activate macrophages and enhance their bactericidal activity [146]. In addition, macrophages and NK cells engage in bidirectional crosstalk that is essential for coordinating effective immune responses against *Mtb*. Upon *Mtb* stimulation, NK cells produce IFN-γ, which induces infected monocytes to secrete IL-15 and IL-18. These cytokines facilitate the expansion and effector function of CD8^+^ IFN-γ^+^ T cells. Importantly, the production of IL-15 and IL-18 by macrophages is dependent on NK-derived IFN-γ, emphasizing the regulatory influence of NK cells on monocyte function. Furthermore, the ability of NK cells to prime CD8^+^ T cells requires direct contact with *Mtb*-infected monocytes and involves CD40L –CD40 interactions. This cellular interplay not only enhances cytokine production by macrophages but also supports the cytolytic activity of CD8^+^ T cells, underscoring the critical role of NK –macrophage cooperation in bridging innate and adaptive immunity during *Mtb* infection [147].

## Strategies to enhance immune cell responses and therapeutic development

Building upon the intricate cellular interactions described earlier, recent research has explored targeted strategies to potentiate immune responses and improve tuberculosis outcomes. Following *Mtb* infection, there is a deficiency in CD40 expression on DCs, which diminishes the interaction with its ligand CD40L on CD4^+^ T cells, leading to a significant reduction in antigen-specific IL-17 responses. In ex vivo T cell-DC co-culture assays, activation of CD40 on DCs with a multimeric CD40 agonist (CD40LT) significantly restores the generation of antigen-specific IL-17. In the context of mucosal DC transfer, augmenting the CD40-CD40L interaction promotes balanced Th1/Th17 responses, and upon aerosol challenge with *Mtb* in mice, it confers enhanced control over pulmonary bacterial loads [148]. Utilizing an agonistic anti-CD40 antibody, researchers induced maturation of bone marrow-derived DCs, enhancing their cytokine production, including IL-1, IL-6, IL-12, and TNF-α. This stimulation amplified the DCs’ ability to activate Th1 responses, characterized by increased IL-12 and IFN-γ levels. Despite these improvements, the enhanced immune response did not translate into increased protection against *Mtb* in vivo, potentially due to insufficient IFN-γ production or the suppressive effects of IL-10. The findings suggest that while CD40 stimulation can potentiate DC function and Th1 responses, additional strategies may be necessary to achieve effective protection against tuberculosis [149].

To develop effective combination chemo-immunotherapeutic strategies against *Mtb* infection, it is crucial to understand the interplay between host immune cell responses and the bactericidal effects of antibiotics. Research has demonstrated that CD4^+^ T lymphocyte response to *Mtb* are diminished following standard chemotherapeutic treatment. Repeated immunization with immunodominant peptides derived from key *Mtb* antigens (ESAT-6 and Ag85B) during antibiotic therapy did not confer additional enhancement beyond the bacterial clearance achieved with chemotherapy alone. Similarly, the adoptive transfer of Th1 cells targeting these antigens during treatment, despite successful migration and proliferation of these cells in the lungs, did not improve bacterial clearance. These findings suggest that enhancing CD4^+^ T lymphocyte responses in the early stages of *Mtb* chemotherapy is unlikely to increase the efficacy of treatment [150]. These findings underscore a critical insight: enhancing CD4^+^ T cell responses during early chemotherapy may have limited additive benefit, as the primary role of T cells appears to be containment rather than eradication of *Mtb* [151].

Recent evidence points to qualitative differences in the capacity of T lymphocytes to recognize infected cells, which carries significant implications for vaccine design. *Mtb*-specific CD4^+^ and CD8^+^ T cells exhibit distinct capabilities in recognizing infected macrophages. While CD4^+^ T lymphocytes specific for antigens like Ag85B and ESAT-6 can effectively recognize and respond to *Mtb*-infected macrophages, CD8^+^ T lymphocytes specific for the TB10.4 antigen show limited recognition of these infected cells. This discrepancy suggests that not all *Mtb*-specific T cells can detect infected macrophages, potentially due to factors such as antigen presentation pathways and antigen abundance. The findings imply that vaccine development should focus on identifying antigens that elicit T cell responses capable of recognizing and targeting infected cells effectively, rather than relying solely on immunogenicity [152].

NK cells play a crucial role in host defense against *Mtb*, particularly in immunocompromised individuals lacking robust T cell responses. Therapeutically, enhancing NK cell activity presents a promising adjunctive strategy for tuberculosis control. Cytokines such as IL-2 and IL-12 significantly stimulate NK cell cytotoxicity and IFN-γ production, which, in turn, activate macrophages and promote intracellular bacterial clearance [132,145,153]. Importantly, supplementation with N-acetylcysteine (NAC) elevates intracellular glutathione (GSH) levels, further boosting NK cell effector functions and arresting *Mtb* growth within monocytes; combined cytokine and NAC treatment yields greater antimycobacterial activity than either alone [154–158]. Mechanistically, NK cells deploy cytotoxic molecules – including perforin, granulysin, FasL, and CD40L – to directly induce apoptosis in infected macrophages and destroy intracellular bacteria independently of accessory cells [78,159–162]. Notably, blockade of FasL or CD40L abrogates these protective effects, underscoring their importance in NK-mediated immunity [154]. These findings suggest that therapeutic strategies aimed at restoring or enhancing NK cell function – via cytokine supplementation or GSH modulation – may offer critical benefits in managing multidrug-resistant tuberculosis and HIV/*Mtb* co-infection scenarios [154,156,158].

Macrophage-targeted therapies offer a promising host-directed approach to combat *Mtb* infection by enhancing macrophage-mediated antimicrobial mechanisms. These strategies focus on reprogramming macrophage phenotypes to restore bactericidal functions and leverage host responses for intracellular bacterial clearance. A key mechanism involves reversing the *Mtb*-induced polarization of macrophages towards an anti-inflammatory M2 phenotype, which is driven by mycobacterial modulation of cytokine secretion, epigenetic remodelling (e.g., SET8-dependent methylation and HDAC expression changes), and metabolic shifts favouring oxidative phosphorylation over glycolysis [163–165]. By promoting pro-inflammatory M1 states, characterized by inducible nitric oxide synthase (iNOS) expression and cytokines like IL-12 and TNF-α, these therapies enhance bacterial elimination.

Building on the modulation of macrophage polarization, pharmacological agents and nanoparticle-based systems have shown promise in restoring M1 functionality. Compounds such as MK-2206 (an AKT inhibitor) and sphingosine-1-phosphate (S1P) induce FOXO3-mediated M1 activation, reduce M2 markers, and improve intracellular bacterial clearance [166,167]. Similarly, MDM2 inhibition with agents like nutlin-3 stabilizes p53, promoting apoptosis of infected macrophages to limit *Mtb* survival [168]. These approaches collectively strengthen the host’s immune response by targeting macrophage plasticity.

A pivotal strategy for macrophage-targeted therapies involves leveraging nanoparticle-based delivery systems for host-directed treatments. Recent advances in nanoparticle-based delivery systems have significantly enhanced macrophage-targeted therapies for tuberculosis (TB), offering innovative host-directed strategies to combat *Mtb* while circumventing antibiotic resistance. A notable development involves macrophage membrane-coated nanoparticles loaded with an aggregation-induced emission (AIE)-based photosensitizer (TPE-BT-BBTD), which enable dual-targeting of granulomas and intracellular *Mtb*. Upon NIR-II laser activation, these biomimetic nanoplatforms deliver photothermal therapy with deep-tissue imaging and minimal systemic toxicity, providing precise, resistance-free treatment [169]. Building on this photothermal approach, another study integrates rifampicin delivery with photothermal therapy in a macrophage-targeted nanoparticle system to treat cutaneous TB, enhancing bacterial clearance and inhibiting ferroptosis via Nrf2/HO-1 signaling, thereby reducing *Mtb* burden and tissue damage with low toxicity [170]. Similarly, a dual-targeting nanoplatform combining AIE photothermal agents with macrophage membrane coating achieves precise localization within granulomas, where NIR-II laser activation generates a potent photothermal effect, outperforming conventional antibiotics and minimizing lung tissue damage [171]. Complementing these photothermal strategies, macrophage-targeted isoniazid-selenium nanoparticles (Ison@Man-Se NPs) have been developed to enhance phagolysosomal fusion and autophagy, amplifying host antimicrobial immunity through reactive oxygen species (ROS) and PI3K/Akt/mTOR signaling to synergistically destroy intracellular *Mtb* [172]. Together, these advancements in nanoparticle-based systems, leveraging biomimetic targeting, photothermal therapy, and autophagy induction, represent a transformative, resistance-free paradigm for precise and effective TB therapy.

In conclusion, macrophage-targeted therapies offer a multifaceted approach to enhance antimycobacterial immunity by reprogramming macrophage phenotypes, inducing autophagy, and restoring reactive species production. These strategies, supported by advanced nanoparticle-based and pulmonary delivery systems, collectively enhance the precision and efficacy of host-directed interventions, presenting a transformative paradigm for managing multidrug-resistant tuberculosis with minimal risk of resistance development.

## Future research directions and perspectives

Upon *Mtb* infection, cytotoxic effector cells release specialized cytotoxic granules with varying functional properties (Table 1). These cytotoxic granules possess distinct capabilities, including killing *Mtb*-infected host cells (host cell apoptosis) and directly targeting and eliminating intracellular bacteria. However, it remains unclear whether a single type of cytotoxic granule can simultaneously mediate both host cell cytotoxicity and pathogen clearance, or whether different subsets of granules are required to perform these functions independently. It is possible that specific granules are specialized solely for host cell apoptosis, while others are tailored for direct antibacterial action, and some may serve a dual role. Future studies should aim to define the functional specialization of these granules across various immune cell types. Moreover, understanding the spatial and temporal dynamics of granule release during different stages of infection will provide crucial insights into how cytotoxic responses are fine-tuned in the TB microenvironment. Deciphering these mechanisms is essential for advancing our comprehension of host-pathogen interactions and for informing the design of novel, targeted immunotherapeutic strategies aimed at enhancing immune-mediated clearance of *Mtb*.

**Table 1.** Expression and function of cytokines or bactericidal products in tuberculosis-infected subject

Cytotoxic gene	Cell type	Effect on cell function	PMID
IFN-γ	CD8^+^ T/NK/NKT/γδ T	granule exocytosis and lysis	24126533, 37003521, 39008002, 10408713, 38741147
TNF-α	CD8^+^ T/Mφ/NK/γδ T	modulate IFN-γ production	24126533, 27247233, 37003521, 38741147
IL-1α/β	DC	anti-bacterial function	22195750, 36591308
IL-2	CD4^+^ T	Cell proliferation	38734321, 37695687
IL-12	DC	Pro-inflammation	28767693
IL-27	Mφ	Pro-inflammation	18557702
NOS2	Mφ	damage microbial components	27866220
Cathelicidin	Mφ	antimicrobial activity	21998409, 36405761
DEFB4	Mφ	antimicrobial activity	25143364, 23289765
PRF1	CD4^+^/CD8^+^T/NK/NKT/ γδ T	killing mycobacteria	37003521, 25139289, 16873912, 19079582, 10408713, 39921673
PRF2	Neu, Mφ, B cells	killing mycobacteria	28422754
Granulysin	NK/CD8^+^ T/γδ T	killing mycobacteria	25139289, 26181627, 37003521, 39921673
GZMA	CD4^+^/CD8^+^ T/NK/γδ T	Cytotoxic function	37003521, 37942326, 23326234
GZMB	CD4^+^/CD8^+^ T/NK/MAIT NKT/γδ T	Cytotoxic function	37003521, 33529407, 10408713
GZMH	NK/γδ T	Cytotoxic function	37003521, 37942326
GZMK	CD8^+^ T	Cytotoxic function	38642678, 36796469
GZMM	γδ T	Cytotoxic function	37942326
CTSW CST7 FCGR3A KSP37 Casp3	NK NK, Mφ NK CD8^+^ T CD4^+^/CD8^+^ T/γδ T/Mφ	Cytolytic activity Inhibits cysteine cathepsins ADCC cytotoxic lymphocyte immunity executor of apoptosis	37003521, 16916327 37003521, 39125711 37174624, 20439102, 24250791 23249543, 23249609 37003521, 36857964, 29983703

Abbreviation: ADCC: Antibody Dependent Cellular mediated Cytotoxicity; ATB: active tuberculosis; Casp3: caspase 3; CST7: Cystatin F; CTSW: Cathepsin W; DC: dendritic cell; DEFB4: Defensin Beta 4A; GZMA: Granzyme A; GZMB: Granzyme B; GZMH: Granzyme H; GZMK: Granzyme K; GZMM: Granzyme M; KSP37: killer-specific secretory protein of 37kDa; Mφ: Macrophage; Neu: Neutrophil; PRF1: Perforin 1; PRF2: Perforin 2 (MPEG1)

Excessive release of these cytotoxic granules can lead to detrimental effects, including the apoptosis of immune cells themselves and the destruction of extracellular matrix components. This, in turn, can result in granuloma rupture, leading to the release of large quantities of *Mtb* previously contained within the granuloma and subsequently triggering the re-dissemination and spread of the pathogen. Therefore, an optimal immune response must strike a delicate balance between effective bacterial killing and preservation of tissue integrity. Elucidating the mechanisms underlying the release and the temporal characteristics of these cytotoxic granules is of significant clinical importance. Such insights could enable precise clearance of *Mtb* without promoting granuloma rupture and pathogen dissemination, thereby enhancing the efficacy of therapeutic interventions targeting tuberculosis.

In addition, future work should explore the potential of modulating cytotoxic granule activity in combination with conventional or host-directed therapies, to augment bacterial clearance while minimizing tissue damage. For example, identifying molecular checkpoints that regulate granule release may offer therapeutic targets to control immune-mediated pathology without compromising host defense. Ultimately, integrating these mechanistic insights into systems-level approaches will be instrumental in developing next-generation TB immunotherapies that are both effective and safe.

## Data Availability

No data was used for the research described in the article.
